# Tamoxifen Twists Again: On and Off-Targets in Macrophages and Infections

**DOI:** 10.3389/fphar.2022.879020

**Published:** 2022-03-30

**Authors:** Chiara Sfogliarini, Giovanna Pepe, Arianna Dolce, Sara Della Torre, Maria Candida Cesta, Marcello Allegretti, Massimo Locati, Elisabetta Vegeto

**Affiliations:** ^1^ Department of Pharmaceutical Sciences, University of Milan, Milan, Italy; ^2^ Dompé Farmaceutici S.p.A., L’Aquila, Italy; ^3^ IRCCS Humanitas Research Hospital, Rozzano, Italy; ^4^ Department of Medical Biotechnologies and Translational Medicine, University of Milan, Milan, Italy

**Keywords:** tamoxifen, SERMs, macrophages, infections, drug repurposing

## Abstract

Beyond the wide use of tamoxifen in breast cancer chemotherapy due to its estrogen receptor antagonist activity, this drug is being assayed in repurposing strategies against a number of microbial infections. We conducted a literature search on the evidence related with tamoxifen activity in macrophages, since these immune cells participate as a first line-defense against pathogen invasion. Consistent data indicate the existence of estrogen receptor-independent targets of tamoxifen in macrophages that include lipid mediators and signaling pathways, such as NRF2 and caspase-1, which allow these cells to undergo phenotypic adaptation and potentiate the inflammatory response, without the induction of cell death. Thus, these lines of evidence suggest that the widespread antimicrobial activity of this drug can be ascribed, at least in part, to the potentiation of the host innate immunity. This widens our understanding of the pharmacological activity of tamoxifen with relevant therapeutic implications for infections and other clinical indications that may benefit from the immunomodulatory effects of this drug.

## Introduction

Tamoxifen is widely used in the treatment of breast cancer for its anti-proliferative activity mediated by the inhibition of estrogen signaling (Patel and Bihani 2018). The decades-long clinical experience along with a favorable pharmacokinetic profile lead to tamoxifen exploitation against other proliferation-related pathologies, such as estrogen-unrelated cancer and microbial infections (Martinez de Dueñas et al., 2014; Montoya and Krysan 2018; Clifford et al., 2020; Su et al., 2021). These off-target indications pertain repurposing strategies that make use of drugs already on the market for other clinical indications, in order to meet the urgent need of novel therapeutics without the expensive and long lasting process of drug discovery (Gil-Gil et al., 2019; Rana et al., 2019; Sharma et al., 2020); of interest to this review is the use of tamoxifen to overcome antimicrobial drug resistance and fight against new infectious diseases.

Beyond estrogen interference and antiproliferative activity, mounting evidence highlight molecular and cellular responses that implicate the involvement of host immune cells. Since macrophages are key immune cells in the fight against cancer and pathogen invasion (Sica et al., 2015), using “tamoxifen” AND “macrophages”, “immunity”, “off-target effects”, “infections”, “repurposing” as keywords, we conducted a systematic search through the PubMed electronic database of all types of studies and collected evidence, according to scientific impact and clinical relevance, on the immunomodulatory activity of tamoxifen in macrophages and infections.

## Molecular and Cellular Mechanisms of Tamoxifen Action

### On and Off-Targets

Orally administered tamoxifen undergoes hepatic conversion into active metabolites, such as 4-hydroxytamoxifen (4HT), that compete with the endogenous sex steroid hormones, estrogens, for binding to intracellular receptors, estrogen receptor alpha (ER*α*) and beta (ER*β*). In fact, 4HT interacts with ERs with a similar binding affinity as the endogenous estrogen, 17*β*-estradiol (E_2_), whereas the prodrug shows a 100-fold lower affinity than 4HT (Rich et al., 2002). ERs are transcription factors that undergo conformational modifications, upon estrogen binding, that induce the receptor to interact with DNA elements within target gene promoters and transcriptional coregulators. Rapid cytoplasmic responses may also be induced by ERs or by estrogen binding to a membrane associated receptor, the G protein-coupled estrogen receptor (GPER), to which 4HT binds with a 1.000-fold lower affinity than ERs (Prossnitz and Arterburn 2015).

Tamoxifen and other ER ligands are defined as selective estrogen receptor modulators (SERMs), in that they induce tissue-selective ER agonist or antagonist effects depending on the interaction with tissue-selective transcriptional coregulators that are recruited by each ligand-specific receptor conformation (McDonnell et al., 2021). As a result, tamoxifen is clinically used in ER*α*-positive breast cancers as an antagonist of estrogen signaling in mammary epithelial cells, yet it provides secondary ER*α*-agonist effects in bone, preventing osteoporosis (Rachner et al., 2018), and in endometrium, leading to an increased risk of endometrial cancer (Braun, Overbeek-Wager, and Grumbo 2016).

Importantly, compelling evidence points to “off-target” responses to the prodrug, tamoxifen, which are mediated by ERα-unrelated, low-affinity effectors described in various cell lineages and physio-pathological conditions. Candidate mediators include PKC (protein kinase C), the transcription factors PPAR*γ* (peroxisome proliferator-activated receptor gamma), GR (glucocorticoid receptor), STAT1 (signal transducer and activator of transcription 1) and NRF2 (nuclear factor erythroid 2-related factor 2) as well as other undefined targets that regulate calcium homeostasis or lipid and sphingolipid metabolism (Mésange et al., 2002; Kim et al., 2008; Matsuoka et al., 2009; Jiang et al., 2013; Bekele et al., 2016; Feng et al., 2017; Hasegawa et al., 2018; Govindarajah et al., 2019; Clifford et al., 2020).

**FIGURE 1:**
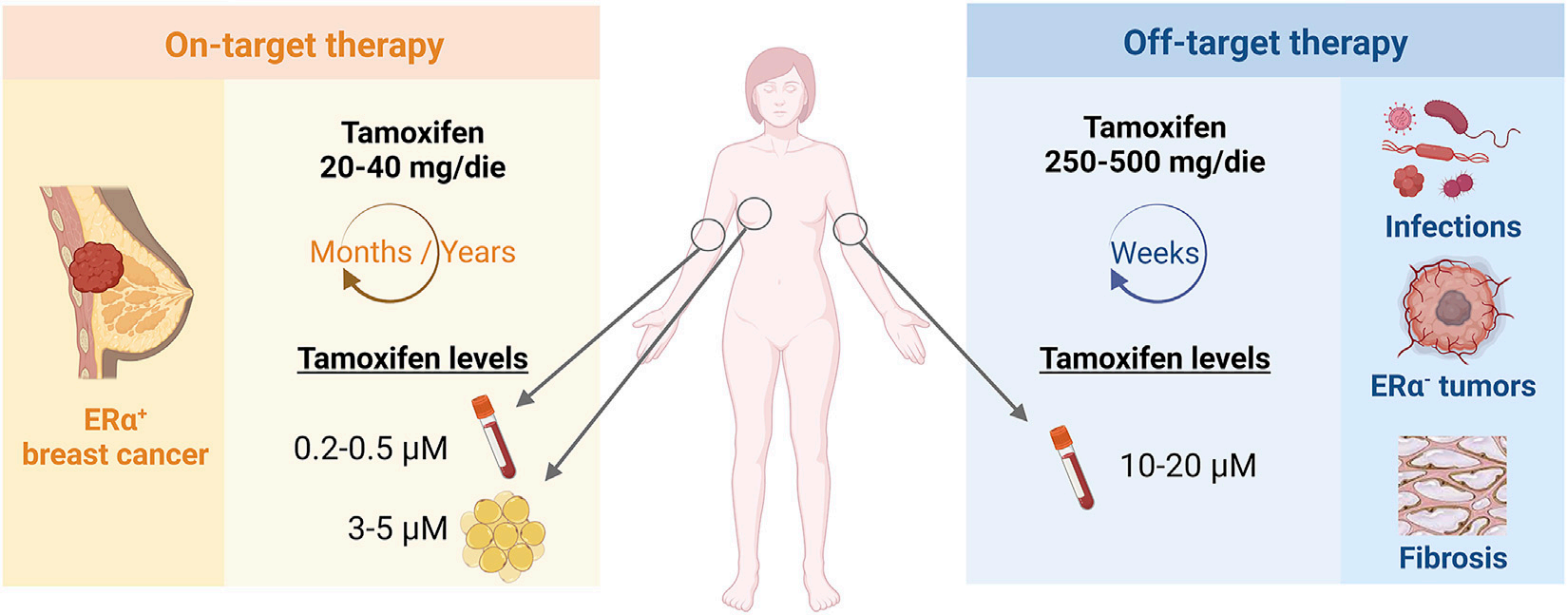
Tamoxifen therapeutic regimen according to on- or off-target indications. Tamoxifen is administered at different concentrations and timings according to on-target, ER*α*-dependent or off-target, ER*α*-unrelated indications and may reach micromolar drug concentrations in patient plasma or tissues.

In clinical practice, the existence of on and off-biological targets translates into distinct tamoxifen therapeutic regimens according to the clinical indication (Figure 1). In the case of ER*α*-positive breast cancer, tamoxifen is prescribed at the daily dose of 20–40 mg/die for years-long treatments. On the other hand, 250–500 mg/die and short-term therapies are prescribed in ER*α*-independent conditions, such as microbial infections or other ERα-negative oncological or fibrotic diseases (Skapek et al., 2013; Odia et al., 2015; Quast et al., 2016; Ngan et al., 2019). Thus, micromolar concentrations of tamoxifen are reached within the tumour mass, as consequence of drug accumulation in the mammary adipose tissue with chronic low-dose regimens, and in the blood of patients undergoing short-term tamoxifen therapy for ERα-independent conditions (Trump et al., 1992; Kisanga et al., 2004; MacCallum et al., 2000). In other words, both chronic and acute pharmacological settings provide high tamoxifen concentrations that supposedly engage low-affinity molecular targets to accomplish the overall therapeutic success.

### Antiproliferative and Oxidative Stress Responses

Cell toxicity induced by high tamoxifen concentrations was originally observed in epithelial cells and associated with inhibition of cell proliferation and induction of apoptosis by estrogen-independent mechanisms, later extended also to non-epithelial cells, such as hepatocytes, fibroblasts and retinal cells (Cho et al., 2012; Yokoyama et al., 2018). These observations led to exploit tamoxifen-induced cytotoxicity in ER*α*-negative cancers, as well as in infections, envisioning a direct pathogen-killing effect of the drug, and fibrosis, for tamoxifen inhibitory effect on proliferation and activity of renal and peritoneal fibroblasts (Vaglio et al., 2006; Dellê et al., 2012; Ma et al., 2015; Wang et al., 2018).

The mechanism of action of tamoxifen cytotoxicity when used at concentrations above the nanomolar range has been linked to the induction of oxidative stress, through the engagement of ER*α*-unrelated, undefined mediators that activate oxidative enzymes or modulate Ca^2+^ homeostasis, increasing the intracellular levels of reactive oxygen species (ROS) or oxidized molecules (Lee et al., 2000; Nazarewicz et al., 2007). Recent molecular studies ascribed tamoxifen responses to the activation of the redox sensitive molecule NRF2, a transcription factor that regulates the expression of antioxidant proteins and promotes cell survival, with interesting implications for antineoplastic drug resistance mechanisms (Itoh et al., 1999; Kim et al., 2008; Bekele et al., 2016). Consistent evidence also indicates activation of inflammasomes and caspases as toxic responses to tamoxifen mediated by oxidative damage and subsequent activation of autophagy and cell apoptosis (Bowie et al., 2004; Cho et al., 2012).

## The Antimicrobial Activity of Tamoxifen

Tamoxifen is effective against a broad range of life-threatening infections. As reported in Table 1, compelling evidence shows that tamoxifen counteracts the proliferation of bacterial species, a wide spectrum of fungal pathogens, human parasites and viruses, including the recently emerged species like Ebola, SARS-CoV, MERS-CoV and SARS-CoV-2 viruses (Dyall et al., 2014; Fan et al., 2017; Montoya and Krysan 2018; Imamura et al., 2021; Zu et al., 2021; Allegretti et al., 2022).

**TABLE 1:** Antimicrobial activity of Tamoxifen.

Type of Study	Pathogen	Experimental Model	Tamoxifen Dosage	Tamoxifen Activity	References
*In vitro*	L. amazonensis	Peritoneal macrophages	5–20 µM	Alkalinization of intracellular vacuoles and suppression of parasite infection	Miguel et al. (2007)
	HCV	Huh-7 cells	1 µM	Suppression of viral genome replication	Watashi et al. (2007)
	Candida C. neoformans	Yeast cells	32–64 μg/ml	Disruption of calmodulin-related processes and cell integrity	Dolan et al. (2009)
	S. cerevisiae				
	L. braziliensis L. chagasi	BMDMs	1–9 µM	Abrogation of intracellular infection	Miguel et al. (2009)
	EBOV	VERO E6, HepG2 cells	1–10 µM	Potent inhibition of viral infection	Johansen et al. (2013)
	HCV	Huh-7 cells	0,1–10 µM	Inhibition of multiple steps of viral life cycle	Murakami et al. (2013)
	C. neoformans	J774 macrophage-like cells	8 μg/ml	Reduced viability within macrophages	Butts et al. (2014)
	MERS-CoV	VERO E6 cells	0,1–100 µM	Antiviral effect observed by drug screening	Dyall et al. (2014)
	SARS-CoV				
	M. tubercolosis	RAW 264.7	3–12 μg/ml	Reduced viability within macrophages	Jang et al. (2015)
	EBOV	HepG2, Hela, HEK293T cells	1–15 µM	Cholesterol and Ca^2+^ accumulation, cellular sphingosine reduction and viral infection inhibition	Fan et al. (2017)
	P. falciparum	Parasite culture	10 µM	Substantial changes in sphingolipid biosynthesis	Piňero et al. (2018)
	C. neoformans C. gattii	Fungal isolates	2–16 μg/ml	Anti-cryptococcal effect alone and in combination with amphotericin	Hai et al. (2019)
	EBOV-like virus	Hela cells	0,1–100 µM	Interference with viral infection through Ca^2+^ channel blockade	Penny et al. (2019)
	EBOV ASFV	VERO E6 cells	10 µM	Inhibition of Ca^2+^ channels, accumulation of cholesterol and inhibition of viral infection	Galindo et al. (2021)
	SARS-CoV-2				
	SARS-CoV-2	VERO E6 cells	10 µM	Inhibitory effect on viral infection	Imamura et al. (2021)
	SARS-CoV-2	VERO E6, Caco-2 cells	1–10 µM	Reduction of S protein production	Zu et al. (2021)
*In vivo*	Candida	Mouse	200 mg/kg/day, 7 days, o.g	Decrease of kidney fungal burden	Dolan et al. (2009)
	Leishmania	Mouse, hamster	20 mg/kg/day, 15 days, i.p	Reduction of parasite burden	Miguel et al. (2009)
	C. neoformans	Mouse	200 mg/kg/day, 3 days, o.g	Improvement of fluconazole anti-cryptococcal activity	Butts et al. (2014)
	SARS-CoV-2	Mouse	60 mg/kg/day, 3 days, i.p	Inhibition of viral RNA loads and inflammatory response	Zu et al. (2021)
Clinical studies	L. braziliensis	Patients with cutaneous leishmaniasis	40 mg/day, orally	Improvement of cure rates in combination with standard treatment for leishmaniasis	Machado et al. (2018)
			0.1% cream, topic use		
			20 days		
	C. neoformans	Patients treated with fluconazole and amphotericin B	300 mg/day, orally	Potential synergistic effect with classic antifungal drugs	Ngan et al. (2019)

Summary of evidence on tamoxifen efficacy against a wide spectrum of pathogens.Legend: L. amazonensis/braziliensis/chagasi, Leishmania; HCV, Hepatitis C Virus; C. neoformans/gattii, Cryptococcus; S. cerevisae, Saccharomyces; BMDMs, Bone Marrow-derived Macrophages; EBOV, Ebola Virus; MERS-CoV, Middle East Respiratory Syndrome Coronavirus; SARS-CoV(-2), Severe Acute Respiratory Syndrome Coronavirus (2); P. falciparum, Plasmodium; ASFV, African Swine Fever Virus; o.g., oral gavage; i.p., intraperitoneally.

Importantly, specific pharmacological features of tamoxifen, mainly its safety profile associated with only mild side effects, oral bioavailability and wide tissue distribution, make a short-term administration of tamoxifen more desirable as opposed to classical anti-infective agents, such as the antifungal fluconazole and amphotericin B, endowed with scarce diffusion and a high degree of toxicity (Butts et al., 2014; Hai et al., 2019; Ngan et al., 2019).

Some pathogen-specific targets have been proposed for tamoxifen antimicrobial activity, such as the calmodulin-dependent signaling pathway in yeast and bacterial cells, the sphingolipid biosynthesis in parasites or viral life cycle proteins (Watashi et al., 2007; Dolan et al., 2009; Murakami et al., 2013; Butts et al., 2014; Piñero et al., 2018). Yet, the wide spectrum of antimicrobial activity of this drug hints to a host-mediated protective system able to kill microbial cells, namely innate immunity. Surprisingly, the activity of tamoxifen has been scarcely investigated in innate immune cells, like macrophages, despite the clinical and pharmacological implications of this mechanism of action.

### Tamoxifen Action Against Enveloped Viruses

Studies on enveloped viruses provided clear evidence for host-mediated, anti-infective actions of tamoxifen and insights into the molecular mechanisms that might also pertain a wider spectrum of anti-microbial activities. Enveloped viruses are endowed with a lipid coating, made of virus-encoded proteins and host-derived lipid membranes. These viral proteins allow the interaction with specific plasma membrane receptors and viral entry within host cells, where viruses are processed from early to late endosomal and, eventually, lysosomal pathways. However, in productive infections, the concerted action of endolysosomal enzymes, ion channels and sphingolipids metabolism increases the outflow of calcium ions and the redistribution of cholesterol, inducing structural changes in the viral and host endolysosomal membranes that merge to form a pore through which the viral genome can access to the cellular sites where replication begins (Mercer et al., 2020).

Tamoxifen and other SERMs are able to inhibit the infections of epithelial cells driven by enveloped viruses, such as Ebola and Hepatitis C virus, without affecting viral entry or endolysosome acidification, but interfering with steps of the viral life cycle that occur after binding and internalization (Johansen et al., 2013; Murakami et al., 2013). Drug responses were shown to be independent from ERs, yet the underlying mechanism of action is still unclear. It is known that SERMs act similarly to other drugs, called cationic amphiphilic drugs (CADs). Due to their lipophilic structures, CADs insert in phospholipid bilayers, particularly endolysosomal membranes where their hydrophilic heads become increasingly protonated by the low endosomal pH. As ionized forms, CADs cannot leave endolysosomes and accumulate up to levels that disturb the metabolism and transport of lipids and proteins (Breiden and Sandhoff 2019). Endosomal ceramide metabolism plays a key role in viral infections, with sphingosine formation that increases calcium efflux and induces the fusion of viral-host membranes. Ceramide metabolism takes place within endosomal lipid rafts, which are microdomains enriched with proteins that regulate signal transduction and other cellular functions, that may also represent druggable targets. Accumulation and interactions of CADs within lipid rafts cause the reduction of cellular sphingosine and increase in endo-lysosomal calcium and cholesterol levels, hindering viral genome exit. Therefore, CADs have been proposed as promising drugs for inhibiting viral infections (Salata et al., 2017). Importantly, tamoxifen activity against viral infections, such as Ebola or SARS-CoV-2 infections, has been associated with inhibition of sphingolipid metabolism and alterations in endosomal calcium and cholesterol traffic (Fan et al., 2017; Penny et al., 2019; Galindo et al., 2021). The increase in ceramide formation and reduction of sphingosine levels by tamoxifen has been also widely described in cancer cells (Furlong, Mader, and Hoskin 2006; Chapman et al., 2010; Morad et al., 2013). The underlying mechanism, although still poorly defined, has been reconciled with inhibition of the acid ceramidase enzyme, by cathepsin-mediated proteolysis, and prevention of ceramide glycosylation (Morad and Cabot 2015).

## Macrophages as Cellular Targets of Tamoxifen

The immune activity of tamoxifen has received little attention so far, although the changes in abundance and function of immune cells, reported in clinical studies with tamoxifen, could participate in drug efficacy (Behjati and Frank 2009).

Macrophages are immune cells mainly committed to the orchestration of inflammation and immune responses. By recognizing a limitless number of physio-pathological signals, macrophages rapidly acquire distinct immune phenotypes defined within two activation states, the classical, pro-inflammatory M1 and the alternative, anti-inflammatory M2 phenotypes. These represent two hypothetical and simplistic extremes of a broad spectrum of intermediate phenotypes acquired by these cells, under the influence of the microenvironment (Locati, Curtale, and Mantovani 2020). M1 activation, as induced by viral and bacterial infections, is characterized by the production of ROS and proinflammatory mediators, such as IL-1β (interleukin one beta), TNF-α (tumor necrosis factor alpha) and IL-6 (interleukin 6), which provide a robust microbe-killing activity (Shapouri-Moghaddam et al., 2018). Anti-inflammatory M2 macrophages are activated by Th2 cytokines, produce signals that promote tissue repair, and actively suppress inflammation (Mantovani and Locati 2013).

### Macrophage On and Off-Targets and Responses

It has been shown that GPER and ER*β* do not mediate the off-target macrophage responses to tamoxifen, since macrophages do not express ER*β* and GPER-selective activation elicits different responses as those induced by tamoxifen. Instead, the ER*α*-mediated effects of E_2_ were inhibited by tamoxifen when assayed at 100-fold higher concentrations than nanomolar levels of the physiologic ligand, providing evidence for being an estrogen antagonist in innate immune cells (Pepe et al., 2018, 2021). Importantly, also ER*α*-independent responses were identified when using micromolar concentrations of tamoxifen. The molecular mechanism of tamoxifen activity in macrophages was initially associated with the activation of PKC and transcription factors, such as GR, PPARy and STAT1 (Komi et al., 2001; Jiang et al., 2013). More recently, the PI3K (phosphatidylinositol 3-kinase)/AKT pathway and NRF2 activation have also been proposed as additional immune targets of tamoxifen, increasing the expression of NRF2 target genes, such as Hmox-1 (heme oxygenase 1), or Vegf-a (vascular endothelial growth factor A) and inhibiting other immune polarization markers such as Il-1b or Arg-1 (arginase 1) (Feng et al., 2018; Pepe et al., 2021). This finding is particularly interesting, considering that NRF2 activation in macrophages prompts protective responses against infections through phagocytosis and autophagy of bacterial or viral particles, induction of intracellular detoxification reactions and potentiation of the inflammatory response (Hai et al., 2011; Harvey et al., 2011; Mornata et al., 2020; Furuya et al., 2016; Bewley et al., 2018; Zhao et al., 2014).

**FIGURE 2:**
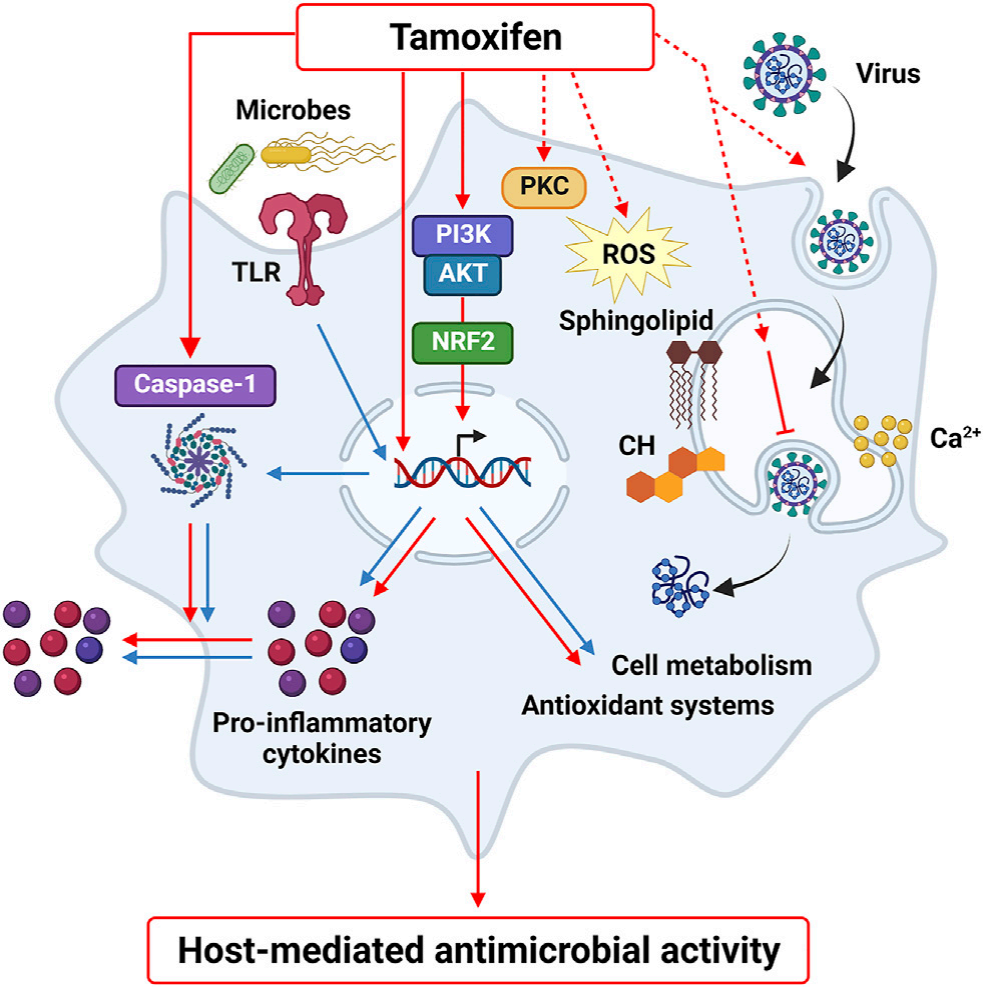
Tamoxifen off-target effects in macrophages. Tamoxifen regulates macrophage activation by inducing PI3K-NRF2 pathway, caspase-1 formation and by modulating lipid metabolism and calcium homeostasis, as well as other possible targets identified in other cell types, such as PKC and oxidative stress. These molecular mediators and cell responses may represent a host-mediated mechanism that contributes to the beneficial activity of tamoxifen against pathogen and viral infections.

Differently from cytotoxicity in epithelial or hepatic cells, off-target effects of tamoxifen do not induce macrophage cell death. Earlier studies reported the influence of tamoxifen on cholesterol homeostasis and lipid metabolism of macrophages, supporting the beneficial consequences of this drug against foam cells formation and atherosclerosis (Dong et al., 2011; Fernández-Suárez et al., 2016; Jiang et al., 2013; Yu et al., 2016). Notably, drug activity in macrophages has been recently extended to immune-metabolic responses. In fact, tamoxifen was shown to potentiate the M1 phenotype, increase phagocytosis and induce active caspase-1 formation (Pepe et al., 2021). Transformation of caspase-1 precursor into the active enzyme promotes IL-1β maturation and secretion by macrophages, thus controlling pathogen infections (Man, Karki, and Kanneganti 2017). Caspase-1 activation may eventually associate with cell death programs; however, IL-1β secretion can occur also independently from cell lysis (Evavold et al., 2018). This latter mechanism has been observed for tamoxifen, which induces active caspase-1 formation in macrophages without affecting cell viability (Pepe et al., 2021). A summary of the possible targets proposed by molecular and cellular studies for tamoxifen activity in macrophages is reported in Figure 2. Whether this immunoregulatory activity underlies the widespread antimicrobial effects of tamoxifen is still unknown. Some data report the efficacy of this drug against the growth of pathogens that, interestingly, proliferate inside macrophages, as in the case of *Salmonella*, Mycobacteria spp and Cryptococcus neoformans (Miguel et al., 2009; Butts et al., 2014; Jang et al., 2015; Lim et al., 2018; Hai et al., 2019; Ngan et al., 2019). Tamoxifen was shown to modify the pH on intracellular vacuoles in infected macrophages, with a beneficial effect in controlling the infection by intracellular parasites such as Leishmania. (Miguel et al., 2007). This suggests that the immunomodulatory effects of this drug in macrophages may provide antimicrobial effects in parallel with a direct antiproliferative activity in microbes.

## Conclusion and Future Directions

Available data in the literature suggest that tamoxifen increases the ability of macrophages to activate an inflammatory response and generate a hostile environment for invading microbes. Although still preliminary, these cellular and molecular responses are triggered by concentrations of tamoxifen that are ensured in the clinical practice by therapeutic regimens that use higher, yet safe and well tolerated, dosages than those used in breast cancer (Skapek et al., 2013; Odia et al., 2015; Ngan et al., 2019). These data strongly support the hypothesis that the immune-mediated effects of tamoxifen contribute to the efficacy of this drug against a wide spectrum of pathogen infections and sustain tamoxifen repurposing in infectious diseases, alone or in combination with standard treatments (Machado et al., 2018; Ngan et al., 2019; Ribeiro et al., 2021).

Importantly, some specific mediators of the off-target effects of tamoxifen have been identified, such as NRF2 signaling, caspase-1 activation and cholesterol redistribution. These pathways are known to control macrophage immunometabolism and inflammatory responses, thus being essential in containing infections. Moreover, tamoxifen was shown to induce neuroprotective effects by regulating microglia activation, further expanding the tissue distribution of drug immune activity (Barreto, Santos-Galindo, and Garcia-Segura 2014; Wu et al., 2021). Therefore, further studies are needed not only to upraise the clinical relevance of tamoxifen activity in macrophages but also to advance target identification and drug development in order to improve therapeutic options. Indeed, the wide availability of drugs like tamoxifen that easily permeate host immune cells, where they trigger beneficial responses, represents a therapeutic advantage for an extensive spectrum of infections, particularly against multidrug-resistant pathogens, where classical therapeutic agents fail, or when infections are driven by intracellular microbes that survive within macrophages. Finally, beyond anti-infective strategies in which tamoxifen repurposing has already proved its efficacy, exploitation of host-mediated responses to tamoxifen will possibly lead to substantial advancement in therapeutic approaches of other proliferation-related pathologies, such as oncological or fibrotic diseases.

## References

[B1] Allegretti M. Cesta M. C. Zippoli M. Beccari A. Talarico C. Mantelli F. (2022). Repurposing the Estrogen Receptor Modulator Raloxifene to Treat SARS-CoV-2 Infection. Cell Death Differ 29 (1), 156–166. 10.1038/s41418-021-00844-6 34404919 PMC8370058

[B2] Barreto G. E. Santos-Galindo M. Garcia-Segura L. M. (2014). Selective Estrogen Receptor Modulators Regulate Reactive Microglia after Penetrating Brain Injury. Front. Aging Neurosci. 6, 132. 10.3389/fnagi.2014.00132 24999330 PMC4064706

[B3] Bekele R. T. Venkatraman G. Liu R. Z. Tang X. Mi S. Benesch M. G. (2016). Oxidative Stress Contributes to the Tamoxifen-Induced Killing of Breast Cancer Cells: Implications for Tamoxifen Therapy and Resistance. Sci. Rep. 6, 21164. 10.1038/srep21164 26883574 PMC4756695

[B4] Bewley M. A. Budd R. C. Ryan E. Cole J. Collini P. Marshall J. (2018). Opsonic Phagocytosis in Chronic Obstructive Pulmonary Disease Is Enhanced by Nrf2 Agonists. Am. J. Respir. Crit. Care Med. 198 (6), 739–750. 10.1164/rccm.201705-0903OC 29547002 PMC6222469

[B5] Bowie M. L. Dietze E. C. Delrow J. Bean G. R. Troch M. M. Marjoram R. J. (2004). Interferon-Regulatory Factor-1 Is Critical for Tamoxifen-Mediated Apoptosis in Human Mammary Epithelial Cells. Oncogene 23 (54), 8743–8755. 10.1038/sj.onc.1208120 15467738

[B6] Braun M. M. Overbeek-Wager E. A. Grumbo R. J. (2016). Diagnosis and Management of Endometrial Cancer. Am. Fam. Physician 93 (6), 468–474. www.aafp.org/afp. 26977831

[B7] Breiden B. Sandhoff K. (2019). Emerging Mechanisms of Drug-Induced Phospholipidosis. Biol. Chem. 401 (1), 31–46. 10.1515/hsz-2019-0270 31408430

[B8] Butts A. Koselny K. Chabrier-Roselló Y. Semighini C. P. Brown J. C. Wang X. (2014). Estrogen Receptor Antagonists Are Anti-cryptococcal Agents that Directly Bind EF Hand Proteins and Synergize with Fluconazole *In Vivo* . mBio 5 (1), e00765–13. 10.1128/mBio.00765-13 24520056 PMC3950514

[B9] Chapman J. V. Gouazé-Andersson V. Messner M. C. Flowers M. Karimi R. Kester M. (2010). Metabolism of Short-Chain Ceramide by Human Cancer Cells--Implications for Therapeutic Approaches. Biochem. Pharmacol. 80 (3), 308–315. 10.1016/j.bcp.2010.04.001 20385104 PMC2883648

[B10] Cho K. S. Yoon Y. H. Choi J. A. Lee S.-J. Koh J.-Y. (2012). Induction of Autophagy and Cell Death by Tamoxifen in Cultured Retinal Pigment Epithelial and Photoreceptor Cells. Invest. Ophthalmol. Vis. Sci. 53 (9), 5344–5353. 10.1167/iovs.12-9827 22786900

[B11] Clifford R. E. Bowden D. Blower E. Kirwan C. C. Vimalachandran D. (2020). Does Tamoxifen Have a Therapeutic Role outside of Breast Cancer? A Systematic Review of the Evidence. Surg. Oncol. 33, 100–107. 10.1016/j.suronc.2020.02.006 32561074

[B12] Dellê H. Rocha J. R. Cavaglieri R. C. Vieira J. M. Jr Malheiros D. M. Noronha I. L. (2012). Antifibrotic Effect of Tamoxifen in a Model of Progressive Renal Disease. J. Am. Soc. Nephrol. 23, 37–48. 10.1681/ASN.2011010046 22052053 PMC3269918

[B13] Dolan K. Montgomery S. Buchheit B. Didone L. Wellington M. Krysan D. J. (2009). Antifungal Activity of Tamoxifen: *In Vitro* and *In Vivo* Activities and Mechanistic Characterization. Antimicrob. Agents Chemother. 53 (8), 3337–3346. 10.1128/AAC.01564-08 19487443 PMC2715577

[B14] Dong P. Xie T. Zhou X. Hu W. Chen Y. Duan Y. (2011). Induction of Macrophage Scavenger Receptor Type BI Expression by Tamoxifen and 4-Hydroxytamoxifen. Atherosclerosis 218 (2), 435–442. 10.1016/j.atherosclerosis.2011.06.048 21820658

[B15] Dyall J. Coleman C. M. Hart B. J. Venkataraman T. Holbrook M. R. Kindrachuk J. (2014). Repurposing of Clinically Developed Drugs for Treatment of Middle East Respiratory Syndrome Coronavirus Infection. Antimicrob. Agents Chemother. 58 (8), 4885–4893. 10.1128/AAC.03036-14 24841273 PMC4136000

[B16] Evavold C. L. Ruan J. Tan Y. Xia S. Wu H. Kagan J. C. (2018). The Pore-Forming Protein Gasdermin D Regulates Interleukin-1 Secretion from Living Macrophages. Immunity 48 (1), 35–e6. e6. 10.1016/j.immuni.2017.11.013 29195811 PMC5773350

[B17] Fan H. Du X. Zhang J. Zheng H. Lu X. Wu Q. (2017). Selective Inhibition of Ebola Entry with Selective Estrogen Receptor Modulators by Disrupting the Endolysosomal Calcium. Sci. Rep. 7, 41226. 10.1038/srep41226 28117364 PMC5259750

[B18] Feng L. Li J. Yang L. Zhu L. Huang X. Zhang S. (2017). Tamoxifen Activates Nrf2-dependent SQSTM1 Transcription to Promote Endometrial Hyperplasia. Theranostics 7 (7), 1890–1900. 10.7150/thno.19135 28638475 PMC5479276

[B19] Feng R. Morine Y. Ikemoto T. Imura S. Iwahashi S. Saito Y. (2018). Nrf2 Activation Drive Macrophages Polarization and Cancer Cell Epithelial-Mesenchymal Transition during Interaction. Cell Commun Signal 16 (1), 54. 10.1186/s12964-018-0262-x 30180849 PMC6122794

[B20] Fernández-Suárez M. E. Escolà-Gil J. C. Pastor O. Dávalos A. Blanco-Vaca F. Lasunción M. A. (2016). Clinically Used Selective Estrogen Receptor Modulators Affect Different Steps of Macrophage-specific Reverse Cholesterol Transport. Sci. Rep. 6, 32105. 10.1038/srep32105 27601313 PMC5013287

[B21] Furlong S. J. Mader J. S. Hoskin D. W. (2006). Lactoferricin-Induced Apoptosis in Estrogen-Nonresponsive MDA-MB-435 Breast Cancer Cells Is Enhanced by C6 Ceramide or Tamoxifen. Oncol. Rep. 15 (5), 1385–1390. 10.3892/or.15.5.1385 16596215

[B22] Furuya A. K. Sharifi H. J. Jellinger R. M. Cristofano P. Shi B. de Noronha C. M. (2016). Sulforaphane Inhibits HIV Infection of Macrophages through Nrf2. Plos Pathog. 12 (4), e1005581. 10.1371/journal.ppat.1005581 27093399 PMC4836681

[B23] Galindo I. Garaigorta U. Lasala F. Cuesta-Geijo M. A. Bueno P. Gil C. (2021). Antiviral Drugs Targeting Endosomal Membrane Proteins Inhibit Distant Animal and Human Pathogenic Viruses. Antivir. Res 186, 104990. 10.1016/j.antiviral.2020.104990 33249093 PMC7690281

[B24] Gil-Gil T. Laborda P. Sanz-García F. Hernando-Amado S. Blanco P. Martínez J. L. (2019). Antimicrobial Resistance: A Multifaceted Problem with Multipronged Solutions. MicrobiologyOpen 8 (11), e945. 10.1002/mbo3.945 31724836 PMC6855134

[B25] Govindarajah N. Clifford R. Bowden D. Sutton P. A. Parsons J. L. Vimalachandran D. (2019). Sphingolipids and Acid Ceramidase as Therapeutic Targets in Cancer Therapy. Crit. Rev. Oncol. Hematol. 138, 104–111. 10.1002/mbo3.94510.1016/j.critrevonc.2019.03.018 31092365

[B84] Hai T. P. Van A. D. Ngan N. Nhat L. Lan N. Vinh Chau N. V. (2011). Targeting Nrf2 Signaling Improves Bacterial Clearance by Alveolar Macrophages in Patients with COPD and in a Mouse Model. Sci. Transl. Med. 3 (78), 78ra32. 10.1126/scitranslmed.3002042 PMC4927975 21490276

[B26] Hai T. P. Van A. D. Ngan N. T. T. Nhat L. T. H. Lan N. P. H. Vinh Chau N. V. (2019). The Combination of Tamoxifen with Amphotericin B, but Not with Fluconazole, Has Synergistic Activity against the Majority of Clinical Isolates of Cryptococcus Neoformans. Mycoses 62 (9), 818–825. 10.1111/myc.12955 31173410 PMC6771715

[B27] Harvey C. J. Thimmulappa R. K. Sethi S. Kong X. Yarmus L. Brown R. H. (2011). Targeting Nrf2 Signaling Improves Bacterial Clearance by Alveolar Macrophages in Patients with COPD and in a Mouse Model. Sci. Transl. Med. 3 (78), 78ra32. 10.1126/scitranslmed.3002042 PMC4927975 21490276

[B28] Hasegawa G. Akatsuka K. Nakashima Y. Yokoe Y. Higo N. Shimonaka M. (2018). Tamoxifen Inhibits the Proliferation of Non-melanoma S-kin C-ancer C-ells by I-ncreasing I-ntracellular C-alcium C-oncentration. Int. J. Oncol. 53 (5), 2157–2166. 10.3892/ijo.2018.4548 30226592

[B29] Imamura K. Sakurai Y. Enami T. Shibukawa R. Nishi Y. Ohta A. (2021). IPSC Screening for Drug Repurposing Identifies Anti-RNA Virus Agents Modulating Host Cell Susceptibility. FEBS open bio 11 (5), 1452–1464. 10.1002/2211-5463.13153 PMC8091584 33822489

[B30] Itoh K. Wakabayashi N. Katoh Y. Ishii T. Igarashi K. Engel J. D. (1999). Keap1 Represses Nuclear Activation of Antioxidant Responsive Elements by Nrf2 through Binding to the Amino-Terminal Neh2 Domain. Genes Dev. 13 (1), 76–86. 10.1101/gad.13.1.76 9887101 PMC316370

[B31] Jang W. S. Kim S. Podder B. Jyoti M. A. Nam K. W. Lee B. E. (2015). Anti-Mycobacterial Activity of Tamoxifen against Drug-Resistant and Intra-macrophage Mycobacterium Tuberculosis. J. Microbiol. Biotechnol. 25 (6), 946–950. 10.4014/jmb.1412.12023 25639719

[B32] Jiang M. Zhang L. Ma X. Hu W. Chen Y. Yu M. (2013). Tamoxifen Inhibits Macrophage FABP4 Expression through the Combined Effects of the GR and PPARγ Pathways. Biochem. J. 454 (3), 467–477. 10.1042/BJ20130580 23805908

[B33] Johansen L. M. Brannan J. M. Delos S. E. Shoemaker C. J. Stossel A. Lear C. (2013). FDA-approved Selective Estrogen Receptor Modulators Inhibit Ebola Virus Infection. Sci. Transl Med. 5 (190), 190ra79. 10.1126/scitranslmed.3005471 PMC3955358 23785035

[B34] Kim S. K. Yang J. W. Kim M. R. Roh S. H. Kim H. G. Lee K. Y. (2008). Increased Expression of Nrf2/ARE-dependent Anti-oxidant Proteins in Tamoxifen-Resistant Breast Cancer Cells. Free Radic. Biol. Med. 45 (4), 537–546. 10.1016/j.freeradbiomed.2008.05.011 18539158

[B35] Kisanga E. R. Gjerde J. Guerrieri-Gonzaga A. Pigatto F. Pesci-Feltri A. Robertson C. (2004). Tamoxifen and Metabolite Concentrations in Serum and Breast Cancer Tissue during Three Dose Regimens in a Randomized Preoperative Trial. Clin. Cancer Res. 10 (7), 2336–2343. 10.1158/1078-0432.ccr-03-0538 15073109

[B36] Komi J. Möttönen M. Luukkainen R. Lassila O. (2001). Non-Steroidal Anti-oestrogens Inhibit the Differentiation of Synovial Macrophages into Dendritic Cells. Rheumatology (Oxford) 40 (2), 185–191. 10.1093/rheumatology/40.2.185 11257155

[B37] Lee Y. S. Kang Y. S. Lee S. H. Kim J. A. (2000). Role of NAD(P)H Oxidase in the Tamoxifen-Induced Generation of Reactive Oxygen Species and Apoptosis in HepG2 Human Hepatoblastoma Cells. Cel Death Differ 7 (10), 925–932. 10.1038/sj.cdd.4400717 11279538

[B38] Lim D. Kim K. S. Jeong J. H. Marques O. Kim H. J. Song M. (2018). The Hepcidin-Ferroportin axis Controls the Iron Content of Salmonella-Containing Vacuoles in Macrophages. Nat. Commun. 9 (1), 2091. 10.1038/s41467-018-04446-8 29844422 PMC5974375

[B39] Locati M. Curtale G. Mantovani A. (2020). Diversity, Mechanisms, and Significance of Macrophage Plasticity. Annu. Rev. Pathol. 15, 123–147. 10.1146/annurev-pathmechdis-012418-012718 31530089 PMC7176483

[B40] Ma G. He J. Yu Y. Xu Y. Yu X. Martinez J. (2015). Tamoxifen Inhibits ER-Negative Breast Cancer Cell Invasion and Metastasis by Accelerating Twist1 Degradation. Int. J. Biol. Sci. 11 (5), 618–628. 10.7150/ijbs.11380 25892968 PMC4400392

[B41] MacCallum J. Cummings J. Dixon J. M. Miller W. R. (2000). Concentrations of Tamoxifen and its Major Metabolites in Hormone Responsive and Resistant Breast Tumours. Br. J. Cancer 82 (10), 1629–1635. 10.1054/bjoc.2000.1120 10817496 PMC2374506

[B42] Machado P. R. L. Ribeiro C. S. França-Costa J. Dourado M. E. F. Trinconi C. T. Yokoyama-Yasunaka J. K. U. (2018). Tamoxifen and Meglumine Antimoniate Combined Therapy in Cutaneous Leishmaniasis Patients: a Randomised Trial. Trop. Med. Int. Health 23 (9), 936–942. 10.1111/tmi.13119 29924907

[B43] Man S. M. Karki R. Kanneganti T. D. (2017). Molecular Mechanisms and Functions of Pyroptosis, Inflammatory Caspases and Inflammasomes in Infectious Diseases. Immunol. Rev. 277 (1), 61–75. 10.1111/imr.12534 28462526 PMC5416822

[B44] Mantovani A. Locati M. (2013). Tumor-Associated Macrophages as a Paradigm of Macrophage Plasticity, Diversity, and Polarization. Arterioscler Thromb. Vasc. Biol. 33 (7), 1478–1483. 10.1161/ATVBAHA.113.300168 23766387

[B45] Martinez de Dueñas E. Ochoa Aranda E. Blancas Lopez-Barajas I. Ferrer Magdalena T. Bandrés Moya F. Chicharro García L. M. (2014). Adjusting the Dose of Tamoxifen in Patients with Early Breast Cancer and CYP2D6 Poor Metabolizer Phenotype. Breast 23 (4), 400–406. 10.1016/j.breast.2014.02.008 24685597

[B46] Matsuoka H. Tsubaki M. Yamazoe Y. Ogaki M. Satou T. Itoh T. (2009). Tamoxifen Inhibits Tumor Cell Invasion and Metastasis in Mouse Melanoma through Suppression of PKC/MEK/ERK and PKC/PI3K/Akt Pathways. Exp. Cel Res 315 (12), 2022–2032. 10.1016/j.yexcr.2009.04.009 19393235

[B47] McDonnell D. P. Wardell S. E. Chang C.-Y. Norris J. D. (2021). Next-Generation Endocrine Therapies for Breast Cancer. Jco 39 (12), 1383–1388. 10.1200/JCO.20.03565 PMC8274744 33705209

[B48] Mercer J. Lee J. E. Saphire E. O. Freeman S. A. (2020). SnapShot: Enveloped Virus Entry. Cell 182 (3), 786–e1. 10.1016/j.cell.2020.06.033 32763187 PMC7409976

[B49] Mésange F. Sebbar M. Capdevielle J. Guillemot J. C. Ferrara P. Bayard F. (2002). Identification of Two Tamoxifen Target Proteins by Photolabeling with 4-(2-Morpholinoethoxy)Benzophenone. Bioconjug. Chem. 13 (4), 766–772. 10.1021/bc015588t 12121132

[B50] Miguel D. C. Yokoyama-Yasunaka J. K. Andreoli W. K. Mortara R. A. Uliana S. R. (2007). Tamoxifen Is Effective against Leishmania and Induces a Rapid Alkalinization of Parasitophorous Vacuoles Harbouring Leishmania (Leishmania) Amazonensis Amastigotes. J. Antimicrob. Chemother. 60 (3), 526–534. 10.1093/jac/dkm219 17584801

[B51] Miguel D. C. Zauli-Nascimento R. C. Yokoyama-Yasunaka J. K. Katz S. Barbiéri C. L. Uliana S. R. (2009). Tamoxifen as a Potential Antileishmanial Agent: Efficacy in the Treatment of Leishmania Braziliensis and Leishmania Chagasi Infections. J. Antimicrob. Chemother. 63 (2), 365–368. 10.1093/jac/dkn509 19095684

[B52] Montoya M. C. Krysan D. J. (2018). Repurposing Estrogen Receptor Antagonists for the Treatment of Infectious Disease. mBio 9 (6), e02272–18. 10.1128/mBio.02272-18 PMC6299222 30563895

[B53] Morad S. A. Cabot M. C. (2015). Tamoxifen Regulation of Sphingolipid Metabolism--Therapeutic Implications. Biochim. Biophys. Acta 1851 (9), 1134–1145. 10.1016/j.bbalip.2015.05.001 25964209 PMC4516673

[B54] Morad S. A. Madigan J. P. Levin J. C. Abdelmageed N. Karimi R. Rosenberg D. W. (2013). Tamoxifen Magnifies Therapeutic Impact of Ceramide in Human Colorectal Cancer Cells Independent of P53. Biochem. Pharmacol. 85 (8), 1057–1065. 10.1016/j.bcp.2013.01.015 23353700 PMC3604153

[B55] Mornata F. Pepe G. Sfogliarini C. Brunialti E. Rovati G. Locati M. (2020). Reciprocal Interference between the NRF2 and LPS Signaling Pathways on the Immune-Metabolic Phenotype of Peritoneal Macrophages. Pharmacol. Res. Perspect. 8 (4), e00638. 10.1002/prp2.638 32794353 PMC7426195

[B56] Murakami Y. Fukasawa M. Kaneko Y. Suzuki T. Wakita T. Fukazawa H. (2013). Selective Estrogen Receptor Modulators Inhibit Hepatitis C Virus Infection at Multiple Steps of the Virus Life Cycle. Microbes Infect. 15 (1), 45–55. 10.1016/j.micinf.2012.10.003 23103222

[B57] Nazarewicz R. R. Zenebe W. J. Parihar A. Larson S. K. Alidema E. Choi J. (2007). Tamoxifen Induces Oxidative Stress and Mitochondrial Apoptosis via Stimulating Mitochondrial Nitric Oxide Synthase. Cancer Res. 67 (3), 1282–1290. 10.1158/0008-5472.CAN-06-3099 17283165

[B58] Ngan N. T. T. Mai N. T. H. Tung N. L. N. Lan N. P. H. Tai L. T. H. Phu N. H. (2019). A Randomized Open Label Trial of Tamoxifen Combined with Amphotericin B and Fluconazole for Cryptococcal Meningitis. Wellcome Open Res. 4, 8. 10.7554/eLife.6892910.12688/wellcomeopenres.15010.1 30801037 PMC6381443

[B59] Odia Y. Kreisl T. N. Aregawi D. Innis E. K. Fine H. A. (2015). A Phase II Trial of Tamoxifen and Bortezomib in Patients with Recurrent Malignant Gliomas. J. Neurooncol. 125 (1), 191–195. 10.1007/s11060-015-1894-y 26285768

[B60] Patel H. K. Bihani T. (2018). Selective Estrogen Receptor Modulators (SERMs) and Selective Estrogen Receptor Degraders (SERDs) in Cancer Treatment. Pharmacol. Ther. 186, 1–24. 10.1016/j.pharmthera.2017.12.012 29289555

[B61] Penny C. J. Vassileva K. Jha A. Yuan Y. Chee X. Yates E. (2019). Mining of Ebola Virus Entry Inhibitors Identifies Approved Drugs as Two-Pore Channel Pore Blockers. Biochim. Biophys. Acta Mol. Cel Res 1866 (7), 1151–1161. 10.1016/j.bbamcr.2018.10.022 PMC7114365 30408544

[B62] Pepe G. Locati M. Della Torre S. Mornata F. Cignarella A. Maggi A. (2018). The Estrogen-Macrophage Interplay in the Homeostasis of the Female Reproductive Tract. Hum. Reprod. Update 24 (6), 652–672. 10.1093/humupd/dmy026 30256960

[B63] Pepe G. Sfogliarini C. Rizzello L. Battaglia G. Pinna C. Rovati G. (2021). Erα-independent NRF2-Mediated Immunoregulatory Activity of Tamoxifen. Biomed. Pharmacother. 144, 112274. 10.1016/j.biopha.2021.112274 34653752

[B64] Piñero T. A. Landoni M. Duschak V. G. Katzin A. M. Couto A. S. (2018). Effect of Tamoxifen on the Sphingolipid Biosynthetic Pathway in the Different Intraerythrocytic Stages of the Apicomplexa Plasmodium Falciparum. Biochem. Biophys. Res. Commun. 497 (4), 1082–1088. 10.1016/j.bbrc.2018.02.183 29496449

[B65] Prossnitz E. R. Arterburn J. B. (2015). International Union of Basic and Clinical Pharmacology. XCVII. G Protein-Coupled Estrogen Receptor and its Pharmacologic Modulators. Pharmacol. Rev. 67 (3), 505–540. 10.1124/pr.114.009712 26023144 PMC4485017

[B66] Quast D. R. Schneider R. Burdzik E. Hoppe S. Möslein G. (2016). Long-Term Outcome of Sporadic and FAP-Associated Desmoid Tumors Treated with High-Dose Selective Estrogen Receptor Modulators and Sulindac: A Single-Center Long-Term Observational Study in 134 Patients. Fam. Cancer 15 (1), 31–40. 10.1007/s10689-015-9830-z 26275868

[B67] Rachner T. D. Coleman R. Hadji P. Hofbauer L. C. (2018). Bone Health during Endocrine Therapy for Cancer. Lancet Diabetes Endocrinol. 6 (11), 901–910. 10.1016/S2213-8587(18)30047-0 29572126

[B68] Rana R. Sharma R. Kumar A. (2019). Repurposing of Existing Statin Drugs for Treatment of Microbial Infections: How Much Promising? Infect. Disord. Drug Targets 19 (3), 224–237. 10.2174/1871526518666180806123230 30081793

[B69] Ribeiro C. S. França R. R. Silva J. A. Silva S. C. D. Uliana S. R. B. Boaventura V. S. (2021). Cellular Infiltrate in Cutaneous Leishmaniasis Lesions and Therapeutic Outcome. Bras Dermatol. 96 (5), 544–550. 10.1016/j.abd.2021.02.006 PMC8441461 34330599

[B70] Rich R. L. Hoth L. R. Geoghegan K. F. Brown T. A. LeMotte P. K. Simons S. P. (2002). Kinetic Analysis of Estrogen Receptor/Ligand Interactions. Proc. Natl. Acad. Sci. U S A. 99 (13), 8562–8567. 10.1073/pnas.142288199 12077320 PMC124311

[B71] Salata C. Calistri A. Parolin C. Baritussio A. Palù G. (2017). Antiviral Activity of Cationic Amphiphilic Drugs. Expert Rev. Anti Infect. Ther. 15 (5), 483–492. 10.1080/14787210.2017.1305888 28286997 PMC7103695

[B72] Shapouri-Moghaddam A. Mohammadian S. Vazini H. Taghadosi M. Esmaeili S. A. Mardani F. (2018). Macrophage Plasticity, Polarization, and Function in Health and Disease. J. Cel Physiol 233 (9), 6425–6440. 10.1002/jcp.26429 PMC13482560 29319160

[B73] Sica A. Erreni M. Allavena P. Porta C. (2015). Macrophage Polarization in Pathology. Cell Mol Life Sci 72 (21), 4111–4126. 10.1007/s00018-015-1995-y 26210152 PMC11113543

[B74] Skapek S. X. Anderson J. R. Hill D. A. Henry D. Spunt S. L. Meyer W. (2013). Safety and Efficacy of High-Dose Tamoxifen and Sulindac for Desmoid Tumor in Children: Results of a Children's Oncology Group (COG) Phase II Study. Pediatr. Blood Cancer 60 (7), 1108–1112. 10.1002/pbc.24457 23281268 PMC4646066

[B75] Su Y. Zhang Y. Hua X. Huang J. Bi X. Xia W. Wang X. Huang Z. Song C. Zhong Y. Shi Y. Wang S. Fan W. Fan W. Yuan Z. South China Breast Cancer Group (SCBCG) (2021). High-Dose Tamoxifen in High-Hormone-Receptor-Expressing Advanced Breast Cancer Patients: A Phase II Pilot Study. Ther. Adv. Med. Oncol. 13, 1758835921993436. 10.1177/1758835921993436 33737962 PMC7934038

[B76] Vaglio A. Salvarani C. Buzio C. (2006). Retroperitoneal Fibrosis. Lancet 367 (9506), 241–251. 10.1016/S0140-6736(06)68035-5 16427494

[B77] Wang S. Li X. Yan L. Nie Q. Dai J. Chen H. (2018). Tamoxifen Inhibits Fibroblast Proliferation and Prevents Epidural Fibrosis by Regulating the AKT Pathway in Rats. Biochem. Biophys. Res. Commun. 497 (4), 937–942. 10.1016/j.bbrc.2018.01.032 29309792

[B78] Watashi K. Inoue D. Hijikata M. Goto K. Aly H. H. Shimotohno K. (2007). Anti-Hepatitis C Virus Activity of Tamoxifen Reveals the Functional Association of Estrogen Receptor with Viral RNA Polymerase NS5B. J. Biol. Chem. 282 (45), 32765–32772. 10.1074/jbc.M704418200 17704057

[B79] Wu S. Y. Sharma S. Wu K. Tyagi A. Zhao D. Deshpande R. P. (2021). Tamoxifen Suppresses Brain Metastasis of Estrogen Receptor-Deficient Breast Cancer by Skewing Microglia Polarization and Enhancing Their Immune Functions. Breast Cancer Res. 23 (1), 35. 10.1186/s13058-021-01412-z 33736709 PMC7977276

[B80] Yokoyama Y. Sasaki Y. Terasaki N. Kawataki T. Takekawa K. Iwase Y. (2018). Comparison of Drug Metabolism and its Related Hepatotoxic Effects in HepaRG, Cryopreserved Human Hepatocytes, and HepG2 Cell Cultures. Biol. Pharm. Bull. 41 (5), 722–732. 10.1248/bpb.b17-00913 29445054

[B81] Yu M. Jiang M. Chen Y. Zhang S. Zhang W. Yang X. (2016). Inhibition of Macrophage CD36 Expression and Cellular Oxidized Low Density Lipoprotein (oxLDL) Accumulation by Tamoxifen: A Peroxisome Proliferator-Activated Receptor (Ppar)Γ-dependent Mechanism. J. Biol. Chem. 291 (33), 16977–16989. 10.1074/jbc.M116.740092 27358406 PMC5016103

[B82] Zhao C. Gillette D. D. Li X. Zhang Z. Wen H. (2014). Nuclear Factor E2-Related Factor-2 (Nrf2) Is Required for NLRP3 and AIM2 Inflammasome Activation. J. Biol. Chem. 289 (24), 17020–17029. 10.1074/jbc.M114.563114 24798340 PMC4059144

[B83] Zu S. Luo D. Li L. Ye Q. Li R. T. Wang Y. (2021). Tamoxifen and Clomiphene Inhibit SARS-CoV-2 Infection by Suppressing Viral Entry. Signal. Transduct Target. Ther. 6 (1), 435. 10.1038/s41392-021-00853-4 34934049 PMC8688909

